# PD-1 and PD-L1 Expression in Inflammatory Bowel Disease: A Paired Immunohistochemical Study

**DOI:** 10.3390/biomedicines14102300

**Published:** 2026-10-10

**Authors:** Harald Bagger-Jörgensen, Christian Thomsen, Klas Sjöberg

**Affiliations:** 1 Department of Clinical Sciences, Malmö, Lund University, 205 02 Malmö, Sweden; klas.sjoberg@med.lu.se; 2 Department of Gastroenterology and Nutrition, Skåne University Hospital, 214 28 Malmö, Sweden; 3 Department of Pathology, Aalborg University Hospital, 9260 Aalborg, Denmark; christian.t@rn.dk; 4 Department of Clinical Medicine, Aalborg University, 9260 Aalborg, Denmark

**Keywords:** Crohn’s disease, inflammation, inflammatory bowel disease, PD-1, PD-L1, ulcerative colitis

## Abstract

**Background/Objectives**: The significance of the PD-1/PD-L1 immune checkpoint pathway in inflammatory bowel disease (IBD) is highlighted by side effects from checkpoint inhibitor therapies including autoimmune enterocolitis reminiscent of IBD. **Methods**: This exploratory study examined PD-1 and PD-L1 expression in active versus inactive IBD in patients with Crohn’s disease (CD) or ulcerative colitis (UC). Immunohistochemical expression was analysed in biopsies obtained during active and inactive IBD from 28 patients (21 UC, 7 CD) using digital image analysis (DIA) and pathologist assessment. Correlations with endoscopic, histological, clinical and laboratory measures were evaluated. **Results**: PD-1 expression was sparse and precluded further statistical analysis. PD-L1 expression was significantly increased in active IBD compared to inactive IBD (*p* < 0.05) in both standard pathologist assessment and DIA. No statistically significant difference in PD-L1 expression was detected between UC and CD, although the small CD subgroup limited the power of this comparison. PD-L1 expression score positively correlated with endoscopic and histopathological inflammatory severity, stool frequency, and daily number of bloody stools. Faecal calprotectin level correlated with PD-L1 expression graded through standard pathologist assessment, but not with DIA. Standard pathologist assessment of PD-L1 expression correlated significantly with DIA PD-L1 scoring. **Conclusions**: In this small exploratory IBD cohort, mucosal PD-L1 expression was higher during active disease and was associated with several measures of inflammatory activity. These findings support further investigation of the PD-1/PD-L1 pathway in intestinal inflammation. DIA provided complementary quantitative assessment of PD-L1 expression.

## 1. Introduction

In the last decade, immune checkpoints have drawn significant attention. T-cell antigen response mediated via the T-cell receptor (TCR) is regulated by inhibitory signals from immune checkpoints and various receptor-ligands belonging to the B7 family expressed on antigen-presenting cells (APCs) and non-lymphoid cells in various organs [1]. Overexpression of immune checkpoint ligands is a major mechanism by which cancer cells develop immune resistance, resulting in the advent of immune checkpoint inhibitors (ICIs), which induce T-cell reactivity to tumour antigens by inhibiting checkpoint pathways. Currently commercially available ICIs specifically target T-lymphocyte-associated protein-4 (CTLA-4) and programmed death 1 (PD-1) pathways. These therapies have become an important part of the oncological treatment arsenal [2] but come with immune-related adverse effects in virtually every organ system, highlighting the role of immune checkpoints in preventing autoimmunity [3]. Gastrointestinal autoimmune side effects are among the most common side effects of ICIs, with up to 13.6% of patients developing enterocolitis [4]. ICI enterocolitis is similar to IBD clinically, endoscopically and histologically, with management empirically based on IBD treatment [5,6]. These findings suggest a role for the immune checkpoint system in the pathophysiology of IBD.

The co-inhibitory PD-1 receptor is found on activated T-cells, activated B-cells, NK-cells, and monocytes in peripheral tissues [7], with its activating ligand PD-L1 being expressed on various organ cells and antigen-presenting cells [8]. PD-L1 expression is typically induced by proinflammatory cytokines such as IFN-γ, TNF-α and IL-6 [9].

Previous studies have investigated the PD-1/PD-L1 pathway in IBD, with one study finding significantly higher expression of PD-1 in colonic mucosal lymphocytes in UC than in CD [10]. One group found a significant increase in PD-L1 expression in colonic mucosal myofibroblasts in UC compared to healthy controls, whereas expression was decreased in CD [11]. However, most studies have been cross-sectional or have compared different patients or different intestinal areas at a single time point. Consequently, changes in mucosal PD-1/PD-L1 expression within the same individual between active and inactive disease remain incompletely characterised.

The aim of this study was to investigate the correlation between colonic PD-1/PD-L1 expression and IBD activity. For this purpose, paired biopsies from 28 patients with IBD were analysed through immunohistochemical analysis of colonic biopsies, correlating expression to IBD severity, assessed through endoscopic and histological inflammation, as well as laboratory and clinical parameters. By using archived biopsy material from a biobank, obtained from the same patients at different time points, we were able to compare PD-1/PD-L1 expression within individuals during active and inactive disease and relate these findings to retrospectively collected measures of disease activity. Although the same patient cohort has previously been used to investigate vitamin D receptor expression [12], the present study instead examined a distinct immunological pathway using separate immunohistochemical analyses.

## 2. Materials and Methods

### 2.1. Patients and Tissue Specimens

Patients with colonic CD or UC attending the in-patient ward or out-patient clinic at the Department of Gastroenterology at Skåne University Hospital, Malmö, between January 2019 and June 2021 were screened for inclusion. Of 491 potentially eligible patients, 57 fulfilled the inclusion criteria, of whom 28 provided written informed consent. The inclusion criteria were at least 2 colonoscopies with biopsy sampling having been performed during the preceding 10 years, in which one must have been in completely inactive disease (defined as no microscopic or endoscopic evidence of inflammation) and one in active disease, with no prior colorectal surgery having taken place.

The Department of Pathology at Region Skåne retains biopsies for 10 years after original evaluation. Thus, two biopsy specimens obtained at separate time points were analysed for each patient: one during active disease and one during inactive disease. The same study population was also used to analyse VDR expression in a separate study [12].

Disease location and behaviour were classified according to the Montreal classification, and IBD medications at the time of each colonoscopy were recorded. When available, clinical symptoms—including abdominal pain, diarrhoea, and bloody stools—as well as laboratory parameters and inflammatory markers (haemoglobin, white blood cell count, albumin, C-reactive protein, and faecal calprotectin) were collected if documented within four weeks of each colonoscopy. Patient characteristics, medication usage, and laboratory findings are presented in Table 1, Table 2 and Table 3.

Macroscopic mucosal inflammation during each colonoscopy was assessed based on the original endoscopic evaluation.

### 2.2. Immunohistochemistry and Histologic Evaluation

Sections 3 µm in thickness were cut from each tissue block and mounted on coated slides at the Department of Pathology, Malmö, Sweden. Two sections were mounted on each slide, representing active and inactive disease states from each patient, respectively. The mounted slides were sent to the Department of Pathology, Aalborg, Denmark.

Slides were baked at 60 °C for at least 30 min and then placed in the automated DAKO Omnis instrument (Agilent) or Ventana Benchmark Ultra (Roche) for PD-L1 and PD-1, respectively. The slides were deparaffinised and submitted to heat-induced epitope retrieval (HIER). PD-L1 slides underwent HIER in Low pH buffer (Agilent) at 99 °C for 40 min, and PD-1 slides in CC1 (Roche) for 48 min. Following endogenous peroxidase blocking, the primary antibodies (PD-L1 mouse monoclonal 22C3, product no. M3653, Agilent, dilution 1:20; PD-1 mouse monoclonal NAT105, product no. 315M-94, Cell Marque, dilution 1:500) were applied for 32 min at 36 °C. After a wash in buffer, the visualisation system (PD-L1: EnVision Flex+, Agilent; PD-1: OptiView DAB, Roche) was applied, and the slides were finally developed with chromogen and counterstained with haematoxylin.

Staining intensity for PD-L1 was scored by a pathologist (A.W.) who was blinded regarding all other information about the samples. The mucosa was scored as negative (0), low (1), moderate (2), or high expression (3). PD-1 staining was qualitatively assessed by another pathologist (C.T.).

The original H&E-stained slides of the biopsies were graded semi-quantitatively by an experienced gastrointestinal pathologist (A.W.) for inflammatory activity according to GI-KVAST (score 0–3) [13].

### 2.3. Digital Image Analysis (DIA)

Slides were scanned at 40× magnification using a Hamamatsu Nanozoomer HT slide scanner. The resulting whole slide images (WSIs) were analysed using QuPath v0.5.1 [14]. Briefly, colour deconvolution was performed on each WSI. Two regions of interest (ROIs) were defined per slide, containing biopsies from the inactive and active time points, respectively. A pixel classifier was applied to distinguish tissue from background, and artefacts such as dirt and folds were manually excluded.

Subsequently, cell detection and object classification were performed to categorise cells as epithelial, stromal, lymphoid, or smooth muscle cells using a pre-trained classifier. H-scores for the epithelial compartment and for the entire mucosa were calculated based on predefined thresholds of cytoplasmic mean DAB optical density (OD). The H-score is a semi-quantitative measure of staining intensity, calculated as the sum of the percentage of cells at each intensity level multiplied by the corresponding intensity score (e.g., 0–3), resulting in a score ranging from 0 to 300.

An experienced pathologist (C.T.) trained the pixel and object classifiers and visually inspected the results to identify any obvious misclassifications.

### 2.4. Statistics

Statistical analyses were performed using SPSS version 27. The paired-samples Wilcoxon test was used to test for statistically significant differences in immunohistochemical PD-1/PD-L1 expression between active and inactive mucosa. The Mann–Whitney U test was used to compare PD-1/PD-L1 expression between UC and CD. Correlations between PD-1/PD-L1 expression, histological inflammation, clinical symptoms, and laboratory measures were determined using Spearman’s coefficient, as all relevant variables were nonparametric. No formal a priori sample-size calculation was performed, as the study included all consenting patients fulfilling the predefined inclusion criteria for whom paired biopsy material was available. No formal adjustment for multiple comparisons was applied. Accordingly, *p*-values from the correlation analyses were nominal and were interpreted as exploratory. Given the limited sample size, particularly the small CD subgroup, and the exploratory nature of the analyses, nonsignificant comparisons between groups were not interpreted as evidence of equivalence.

### 2.5. Ethics

This study was approved by the Swedish Regional Review Authority (Dnr 2021-02219) and Region Skåne Consultative Board for Quality Registers, Healthcare Registers and Preparation (registration number 159-21). Written informed consent was obtained from all patients prior to inclusion.

## 3. Results

### 3.1. Patients and Disease Characteristics

Following the selection process, 28 patients were included. A total of 56 biopsies were thus analysed (2 per patient). Twenty-one patients with UC and seven with colonic CD comprised the study population. Table 1 and Table 2 provide information on the patients’ disease characteristics and IBD medications at the time of each colonoscopy. Basic laboratory tests are shown in Table 3.

### 3.2. PD-1 Expression Limited to Lymphoid Follicles; No Statistically Significant Difference in PD-L1 Staining Intensity Between UC and CD

**Figure 1:**
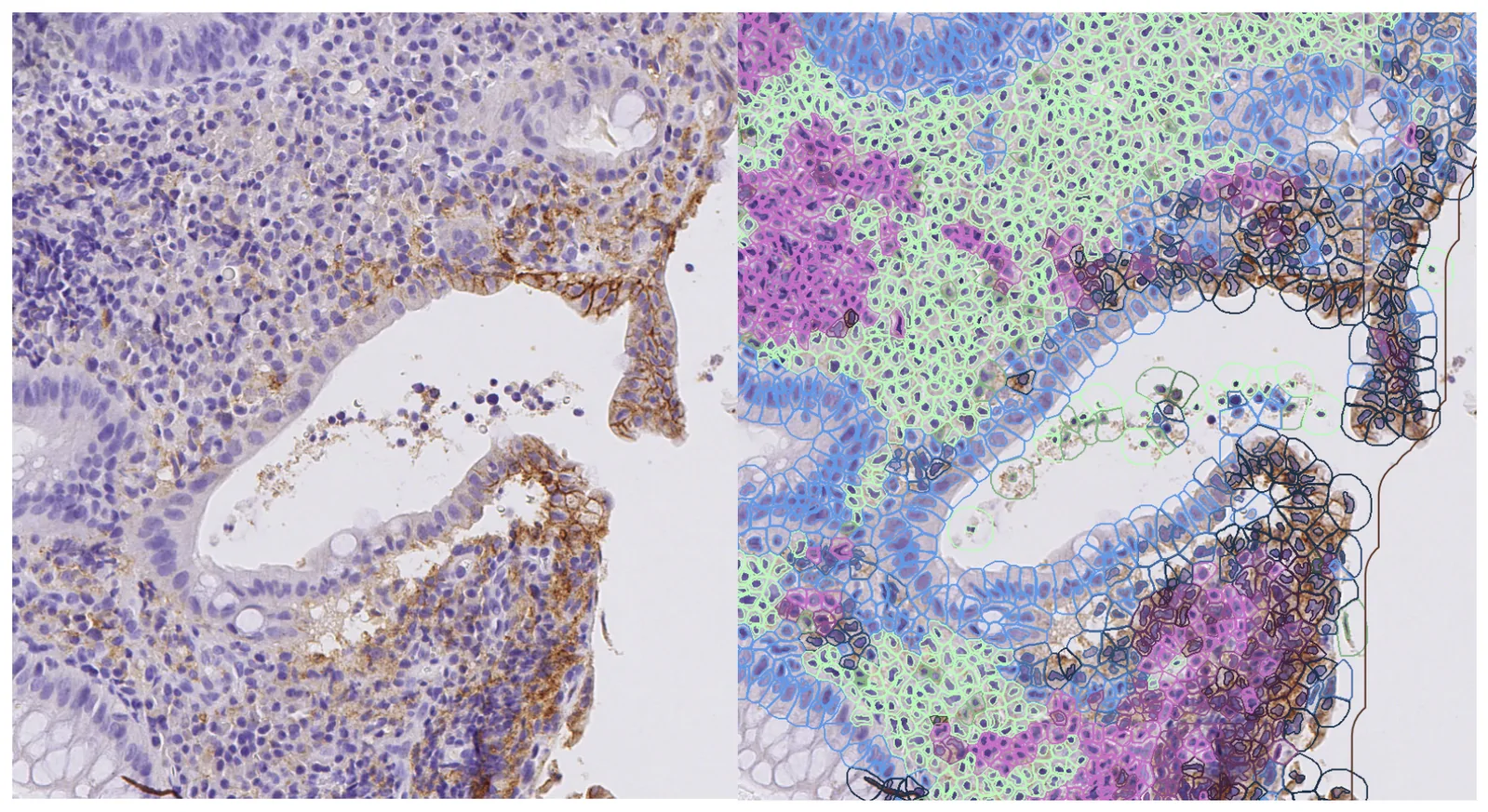
Digital image analysis of PD-L1 immunohistochemistry performed using QuPath. Following cell detection, an object classifier was applied to categorise cells as epithelial (blue), stromal (green), or lymphoid (purple). Staining intensity was quantified based on the mean cytoplasmic DAB (brown) optical density and assigned an intensity score from 0 to 3. This is visualised as a colour gradient ranging from light (score 0) to dark (score 3).

PD-1 expression was only positive in lymphoid follicles with germinal centres, which were present in 6/56 cases, precluding further statistical analysis. PD-L1 stained positively in epithelial, stromal and immune cells. Median PD-L1 staining intensity in active IBD was 1.0 (IQR 0.0–2.0) by standard pathologist assessment and 4.4 (IQR 1.9–13.9) by DIA. In inactive IBD, PD-L1 median staining was 0.0 (IQR 0.0–0.5) by standard pathologist assessment and 1.9 (IQR 1.0–3.0) by DIA. No significant difference in staining intensity was observed between UC and CD in PD-L1 in either standard pathologist assessment (*p* = 0.12) or DIA (*p* = 0.28). Representative PD-L1 immunohistochemistry with DIA is shown in Figure 1.

### 3.3. PD-L1 Expression Correlates with IBD Inflammatory Activity

**Figure 2:**
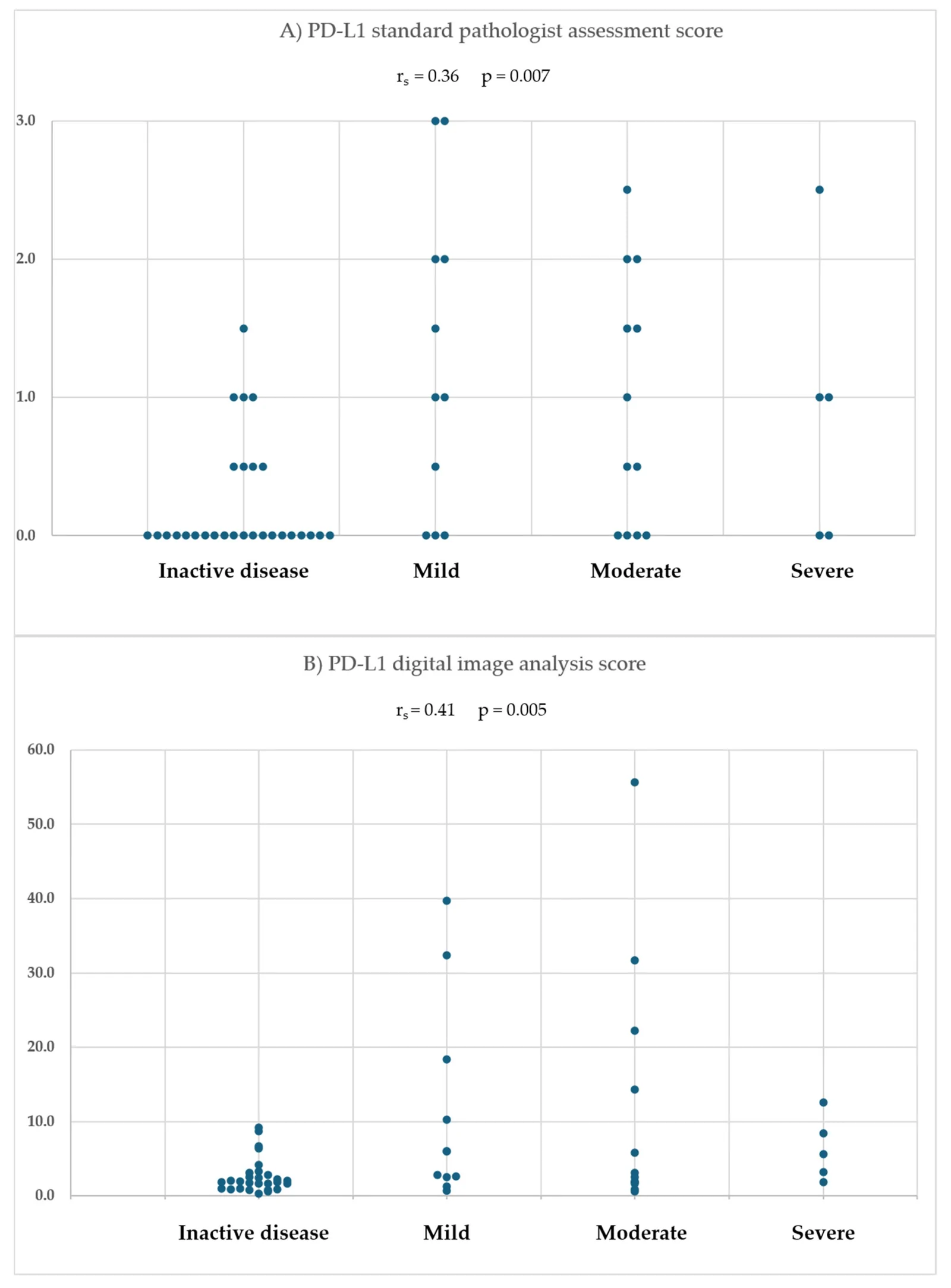
Correlation between histological IBD inflammatory severity and PD-L1 expression assessed by both standard pathologist assessment (**A**) and digital image analysis (**B**).

**Figure 3:**
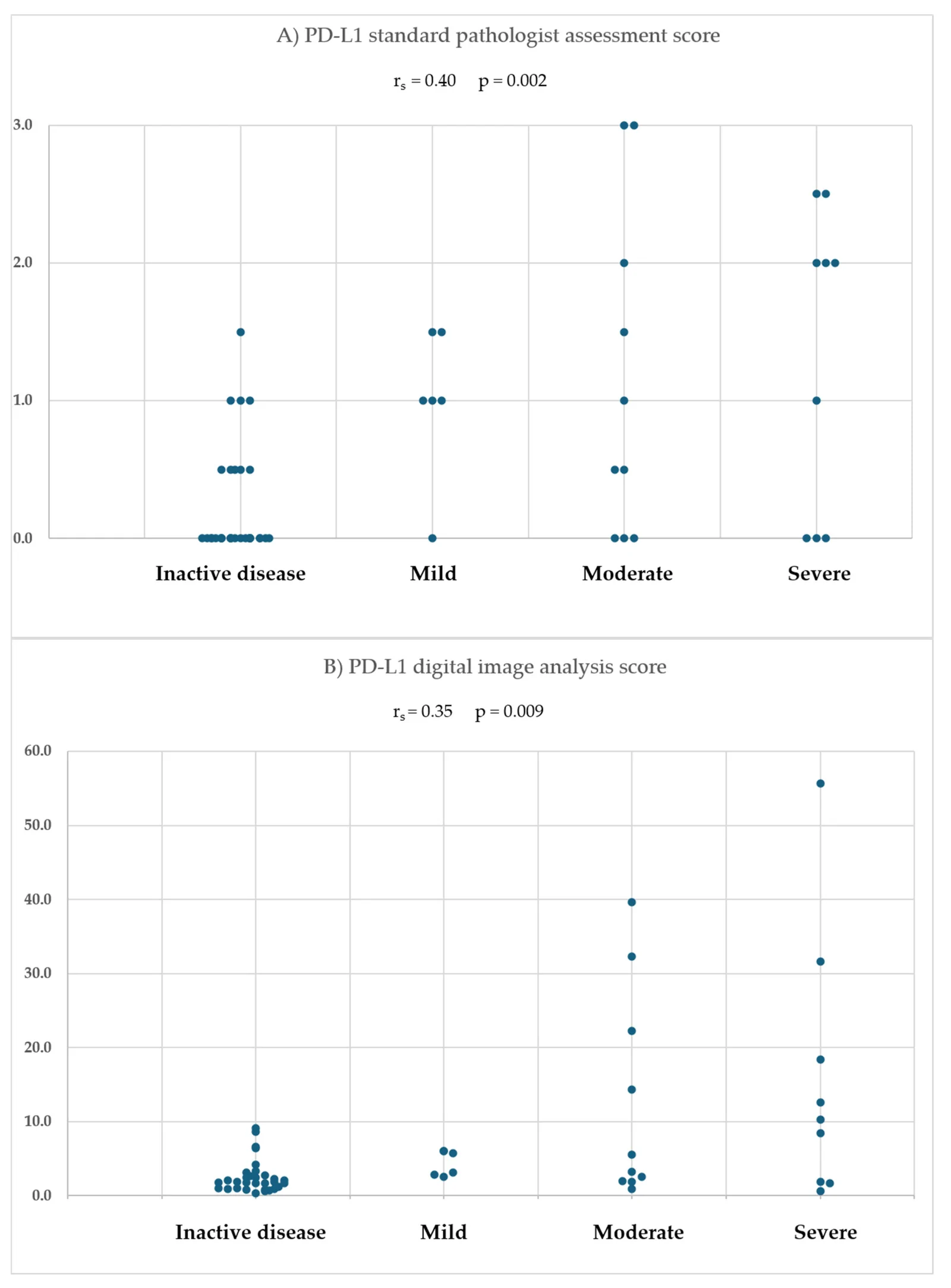
Correlation between endoscopic IBD inflammatory severity and PD-L1 expression assessed by both standard pathologist assessment (**A**) and digital image analysis (**B**).

**Figure 4:**
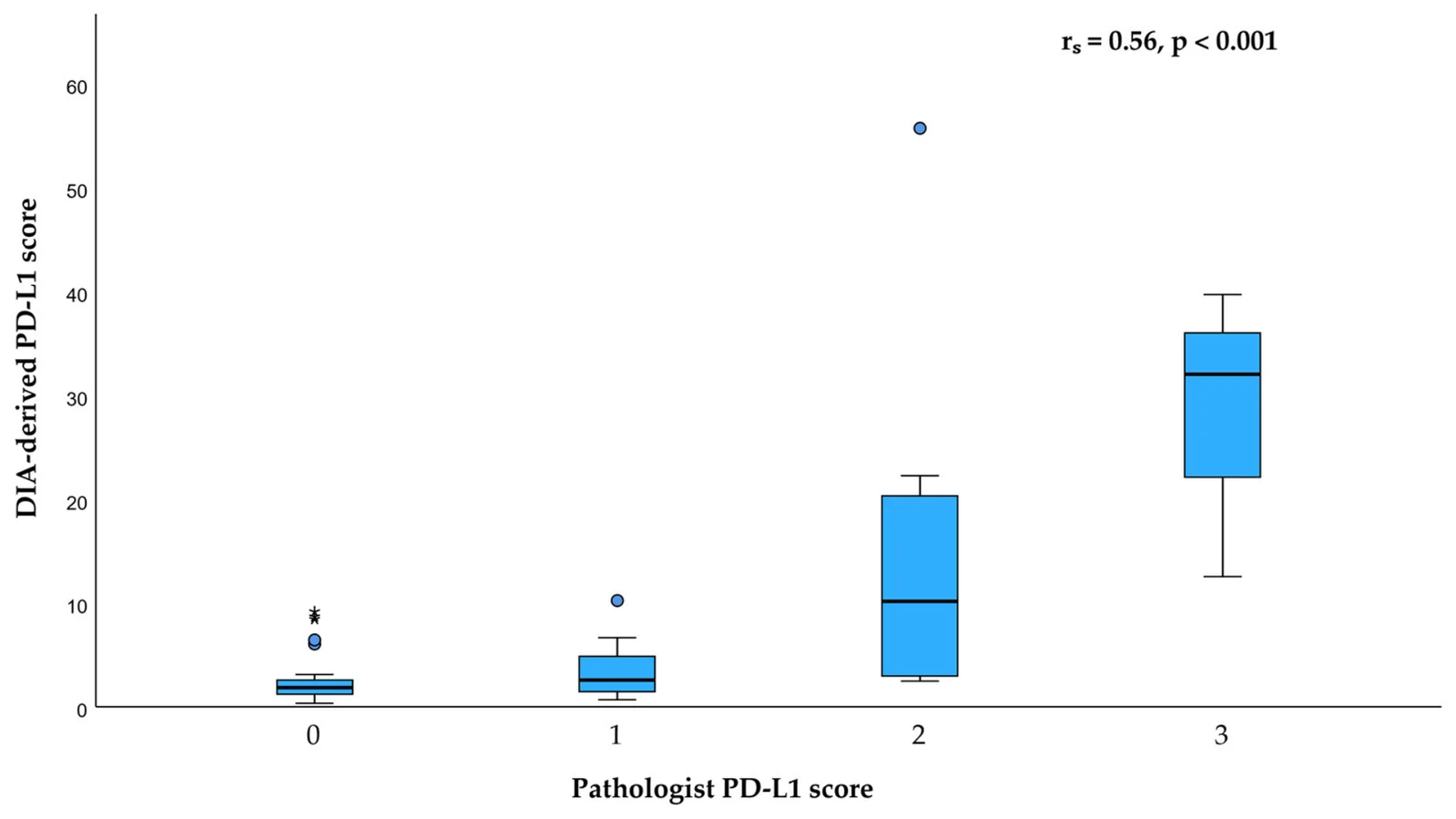
Correlation between standard pathologist assessment and digital image analysis of PD-L1 expression. DIA-derived PD-L1 H-scores are shown according to pathologist PD-L1 staining score (0–3). Pathologist assessment correlated positively with DIA-derived H-score (r_s_ = 0.56, *p* < 0.001). Circles (^◯^) indicate outliers (1.5–3 IQR from the box) and asterisks (*) indicate extreme outliers (>3 IQR from the box).

PD-L1 expression was significantly stronger in active IBD compared to inactive IBD with both standard pathologist assessment (*p* = 0.01) and DIA (*p* = 0.02). PD-L1 expression assessed by standard pathologist scoring correlated positively with DIA-derived H-scores (n = 56, r_s_ = 0.56, *p* < 0.001). PD-L1 staining intensity correlated significantly with histopathological score (n = 56, r_s_ = 0.41, *p* < 0.005 for standard pathologist assessment; n = 56, r_s_ = 0.36, *p* = 0.007 for DIA), endoscopic inflammatory score (n = 56, r_s_ = 0.40, *p* = 0.002 for standard pathologist assessment; n = 56, r_s_ = 0.35, *p* = 0.009 for DIA), stool frequency (n = 55, r_s_ = 0.46, *p* < 0.005 for standard pathologist assessment; n = 55, r_s_ = 0.39, *p* < 0.005 for DIA) and daily number of bloody stools (n = 56, r_s_ = 0.35, *p* = 0.008 for standard pathologist assessment; n = 56, r_s_ = 0.40, *p* < 0.005 for DIA). Also, there was a significant correlation between PD-L1 staining intensity and faecal calprotectin (n = 27, r_s_ = 0.45, *p* = 0.019) with standard pathologist assessment; a similar trend was noted with DIA but did not reach statistical significance (n = 27, r_s_ = 0.33, *p* = 0.09). No other significant correlations were found between PD-L1 and the inflammatory parameters examined. Correlations between PD-L1 expression and histological and endoscopic scores are shown in Figure 2 and Figure 3, respectively. Figure 4 shows the correlation between DIA and standard pathologist-based PD-L1 scoring.

## 4. Discussion

In this study we have demonstrated significantly increased PD-L1 expression in active IBD, with staining intensity correlating with endoscopic and histological inflammation as well as stool frequency. PD-L1 expression also correlated positively with bloody stools and faecal calprotectin levels. No statistically significant difference in PD-L1 expression was detected between UC and CD; however, the small CD subgroup substantially limits conclusions regarding disease-specific differences. Of note, PD-1 was only expressed in immune cells in germinal centres of lymphoid follicles. We used a dual approach to grade PD-L1 immunohistochemical expression using both machine learning-based digital image analysis (DIA) and standard pathologist assessment. DIA provides a more standardised quantitative assessment and may reduce inter- and intra-observer variability. Additionally, it allows for more nuanced assessment in borderline cases (where a four-tiered visual scoring system may be insufficiently sensitive to subtle differences). However, its accuracy depends on the training of algorithms and the ability to identify and correct misclassifications.

In terms of disease characteristics, our patient group was representative of the IBD population [15,16], except for a comparatively higher prevalence of ulcerative pancolitis (67 percent of our UC patients) than the typical relative prevalence of 15–25 percent [16] and a marked predominance of UC (21 out of 28 patients). This is likely due to our inclusion criterion of having undergone multiple colonoscopies in recent years, naturally selecting patients for whom repeated colonoscopy may be indicated, of which ulcerative pancolitis is a typical indication.

Our results are consistent with prior studies demonstrating altered mucosal PD-1/PD-L1 expression in IBD and associations with inflammatory activity [10,11,17,18,19,20,21]. Our findings demonstrate increased mucosal PD-L1 expression in association with inflammatory activity within IBD. This may represent an immunoregulatory response to intestinal inflammation; however, the present observational study cannot prove causality, establish the functional consequences of increased PD-L1 expression, or determine whether this response is specific to IBD.

Some studies have suggested that dysregulation of the PD-1/PD-L1 system plays a role in the pathogenesis of IBD. Rajabian et al. [17] found a significant increase in PD-1/PD-L1 mRNA expression in UC compared to healthy controls. As we did not have a control group, we could not draw such conclusions based on our data. However, PD-1/PD-L1 expression also increases in infectious colitis [10], showing that mucosal induction of the PD-1/PD-L1 system is likely a consequence of any (hyper)inflammatory state in the colon, regardless of aetiology. Notably, increased mucosal PD-L1 expression has also been described in viral infections [9,22].

Whether modulation of the PD-1/PD-L1 pathway could have therapeutic potential in IBD remains speculative, although recent reviews have highlighted checkpoint modulation as an emerging therapeutic strategy in IBD [23]. Experimental studies provide some support for this hypothesis: Song et al. [24] ameliorated experimentally induced murine colitis through administration of PD-L1-Fc, while Yamamoto et al. [25] reported a therapeutic effect following PD-L1 induction with entecavir. However, these experimental observations cannot be extrapolated directly from the present clinical data, and any potential anti-inflammatory benefit would need to be weighed against possible effects on antitumour immune surveillance.

In contrast to Roosenboom et al. [10], who found higher intensity of PD-1 expression in lamina propria in UC compared to CD, and Beswick et al. [11] and Nguyen et al. [19], who reported higher levels of PD-L1 in UC lamina propria mucosa compared to CD, we found no statistically significant difference in PD-L1 staining between UC and CD. We have no clear explanation for this discrepancy. Also, despite using the same anti-PD-1 antibody, we did not observe positivity in lymphoid cells outside germinal centres, which is in marked contrast to their findings. This may be due to differences in antibody concentration and other protocol settings, leading to lower sensitivity in our PD-1 assay. Our assay is used in routine haematopathological diagnostics and is optimised for that use. Roosenboom et al. used a larger sample size and flow cytometry as a complement to immunohistochemistry, which may have enabled the detection of smaller differences. Also, Beswick et al. used an altogether different methodology for PD-L1 measurement, using flow cytometry and immunofluorescence, making their results difficult to compare with ours. Accordingly, our findings should not be interpreted as evidence of equivalent PD-L1 expression in UC and CD.

Our study has several limitations. First, the cohort was small, particularly the CD subgroup, which comprised only seven patients. The study should therefore be considered exploratory, and the absence of statistically significant differences between UC and CD cannot be interpreted as evidence of equivalent PD-L1 expression. Second, the retrospective design resulted in substantial missing clinical and laboratory data, reducing the effective sample size of several analyses. Third, patients received heterogeneous IBD therapies that may influence mucosal immune signalling and PD-L1 expression. The limited sample size and treatment heterogeneity precluded meaningful adjustment for individual therapies, and treatment-related confounding therefore cannot be excluded. Fourth, the absence of a healthy control group means that our findings demonstrate an association between PD-L1 expression and inflammatory activity within IBD but cannot establish an IBD-specific alteration of the PD-1/PD-L1 pathway. Finally, the multiple exploratory correlation analyses increase the risk of type I error and should therefore be interpreted cautiously.

Our study also has some strengths. Prior studies [10,11,17,18,19,20,21] have been cross-sectional, contrasting active and inactive lesions in active disease from the same colonoscopy at a particular point in time, and therefore could not track patients over time in terms of inflammatory activity and PD-1/PD-L1 expression. Although our data were collected retrospectively, the availability of biopsies from separate time points allowed us to assess longitudinal changes in PD-1/PD-L1 expression within individual patients. Thus, despite the absence of a separate control group, each patient served as their own control, allowing direct comparison of PD-1/PD-L1 expression during remission and active disease. Given that we have examined these parameters in the same patient at two distinct times, we believe our within-patient design may provide a more representative assessment of changes in PD-1/PD-L1 expression associated with disease activity.

## 5. Conclusions

In this small exploratory cohort, intestinal mucosal PD-L1 expression was increased during active compared with inactive IBD and was associated with several measures of inflammatory activity. No statistically significant difference in PD-L1 expression was detected between UC and CD, although the small CD subgroup limits conclusions regarding disease-specific differences. These findings support further investigation of the PD-1/PD-L1 pathway in intestinal inflammation in larger, independently recruited cohorts.

## Figures and Tables

**Table 1:** Basic disease characteristics of recruited patients. PSC: primary sclerosing cholangitis, EN: erythema nodosum, PG: pyoderma gangrenosum, HS: hidradenitis suppurativa. This table also features in our previous manuscript [12], which included the same patient population.

Crohn’s Disease		Ulcerative Colitis	
Number (%)	7 (25)		21 (75)
Male: female	4:3		13:8
Age at biopsy, mean (range), years	40 (13–57)		44 (26–81)
Age at diagnosis, mean (range), years	25 (10–46)		33 (14–66)
Disease duration, mean (range) years	13 (0–42)		10 (0–29)
**Location, n (%)**		**Extent, n (%)**	
Colonic	5 (71)	Proctitis	3 (14)
Small intestine and colon	2 (29)	Left sided	4 (19)
		Extensive	14 (67)
**Behaviour, Crohn’s disease**			
Nonstricturing, nonpenetrating	4 (57)		
Stricturing	1 (14)		
Penetrating	2 (29)		
**Prior ileocaecal resection, n (%)**			
Yes	2 (29)		
No	5 (71)		
**Extraintestinal manifestations, n (%)**			
None	2 (29)		12 (57)
Arthralgia	3 (43)		8 (38)
Ocular system (uveitis, episcleritis)	0 (0)		1 (5)
PSC	2 (29)		1 (5)
Skin manifestations (EN, PG, HS)	0 (0)		0 (0)

**Table 2:** Medical therapy in inflammatory bowel disease (IBD) patients. There was some overlap between medication groups; therefore, the sum of percentages in some cases exceeds 100%. This table also features in our previous manuscript [12], which included the same patient population.

	Crohn’s Disease, Active	Crohn’s Disease, Inactive	Ulcerative Colitis, Active	Ulcerative Colitis, Inactive
**IBD medications, n (%)**				
None	4 (57)	1 (14)	4 (19)	1 (5)
5–ASA only	0 (0)	0 (0)	4 (19)	5 (24)
Corticosteroids ± 5–ASA	2 (29)	0 (0)	6 (29)	0 (0)
Azathioprine ± 5–ASA/corticosteroids	0 (0)	0 (0)	3 (14)	4 (19)
Infliximab ± 5–ASA/Azathioprine/corticosteroids	0 (0)	5 (71)	4 (19)	10 (48)
Vedolizumab ± 5–ASA/Azathioprine/corticosteroids	1 (14)	1 (14)	0 (0)	1 (5)
Antibiotics	2 (29)	1 (14)	2 (10)	2 (10)

**Table 3:** Laboratory data from patients. CRP: C-reactive protein, Hb: haemoglobin, WBC: white blood cell count. Values are presented as mean (range) or median (Q1/Q3) after analysis of skewness. This table also features in our previous manuscript [12], which included the same patient population.

	Crohn’s Disease, Active	Crohn’s Disease, Inactive	Ulcerative Colitis, Active	Ulcerative Colitis, Inactive
**Mean (range)**				
Hb, g/L	129 (97–151)	141 (120–162)	132 (91–177)	141 (94–189)
Platelet count, ×10^9^/L	342 (227–564)	267 (167–344)	319 (200–552)	292 (206–517)
Albumin, g/L	34 (25–40)	40 (33–48)	38 (23–45)	41 (25–49)
F–calprotectin, mg/kg	554 (437–670)	185 (157–212)	1021 (180–2260)	166 (45–322)
**Median (Q1–Q3)**				
CRP, mg/L	27.9 (2.1–116.0)	3.9 (1.4–11.0)	3.1 (0.8–12.0)	<0.6 (<0.6–1.6)
WBC, ×10^9^/L	8–9 (4.1–15.5)	6.5 (3.8–9.5)	9.3 (6.0–11.6)	6.2 (3.3–17.0)

## Data Availability

The raw data supporting the conclusions of this article will be made available by the authors on request.
